# One, No One, and One Hundred Thousand: The Many Forms of Ribonucleotides in DNA

**DOI:** 10.3390/ijms21051706

**Published:** 2020-03-02

**Authors:** Giulia Maria Nava, Lavinia Grasso, Sarah Sertic, Achille Pellicioli, Marco Muzi Falconi, Federico Lazzaro

**Affiliations:** Dipartimento di Bioscienze, Università degli Studi di Milano, via Celoria 26, 20133 Milano, Italy; giulia.nava@unimi.it (G.M.N.); lavinia.grasso@unimi.it (L.G.); sarah.sertic@unimi.it (S.S.); achille.pellicioli@unimi.it (A.P.)

**Keywords:** rNMPs incorporation, RNA:DNA hybrids, RNase H, replication stress, genome instability

## Abstract

In the last decade, it has become evident that RNA is frequently found in DNA. It is now well established that single embedded ribonucleoside monophosphates (rNMPs) are primarily introduced by DNA polymerases and that longer stretches of RNA can anneal to DNA, generating RNA:DNA hybrids. Among them, the most studied are R-loops, peculiar three-stranded nucleic acid structures formed upon the re-hybridization of a transcript to its template DNA. In addition, polyribonucleotide chains are synthesized to allow DNA replication priming, double-strand breaks repair, and may as well result from the direct incorporation of consecutive rNMPs by DNA polymerases. The bright side of RNA into DNA is that it contributes to regulating different physiological functions. The dark side, however, is that persistent RNA compromises genome integrity and genome stability. For these reasons, the characterization of all these structures has been under growing investigation. In this review, we discussed the origin of single and multiple ribonucleotides in the genome and in the DNA of organelles, focusing on situations where the aberrant processing of RNA:DNA hybrids may result in multiple rNMPs embedded in DNA. We concluded by providing an overview of the currently available strategies to study the presence of single and multiple ribonucleotides in DNA in vivo.

## 1. Introduction

The presence of single ribonucleotides in DNA has been extensively studied and reported in many excellent reviews [1,2,3,4]; here, we just recalled some important details about their sources, effects, and removal. On the other hand, we still lack a complete understanding of the different types of multiple rNMPs that can be found in DNA. Most of the published literature about RNA:DNA hybrids focus on R-loops, but the world of RNA:DNA hybrids is much wider: it also includes RNA primers found at Okazaki fragments, hybrids formed at double-strand breaks (DSBs), polyribonucleotide stretches eventually incorporated by DNA polymerases, etc. In this review, we thus discussed with particular interest the possible sources and consequences of inserting multiple rNMPs into DNA.

## 2. DNA Polymerases are the Main Source of Single Ribonucleotides Introduced in DNA

### 2.1. DNA Replication

Most leaving organisms store their genetic information in DNA rather than in RNA, partly because of the inherent chemical instability of the RNA molecule. The DNA, indeed, lacks the reactive 2′-OH group on the ribose sugar, which can attack the sugar-phosphate backbone, generating breaks with genotoxic outcomes [5]. The DNA must, therefore, be carefully duplicated for proper transmission of the genetic information over many generations, avoiding mutations that can promote genome instability and related human pathologies, like cancer or neurodegenerative diseases [6,7]. The accuracy of DNA replication is ensured not only by the high-fidelity rate of replicative DNA polymerases and their associated proofreading activities but also by numerous other replicative and post-replicative factors and mechanisms, including DNA repair systems [8,9]. Apart from choosing the proper complementary base, replicative DNA polymerases must also discriminate between sugars, ribose in rNTPs versus deoxyribose in dNTPs [10]. This is why replicative DNA polymerases, like most DNA polymerases, are equipped with a special “steric-gate” residue localized in their nucleotide-binding pocket. Steric-gate residues (Tyrosine or Phenylalanine in B-family polymerases) are characterized by a bulky side chain that sterically clashes with the 2′-OH on the ribose ring of incoming rNTPs, thus preventing their incorporation in DNA [11]. Other active site residues are as well necessary to keep the side chain of the steric-gate residue and the incoming nucleotide in the proper orientation to achieve high sugar selectivity; for example, the backbone NH of a highly conserved hydrophobic residue flanking the N-terminus of the steric-gate residue can form a hydrogen bond with a non-bridging oxygen in the β-phosphate of a bound nucleotide [11]. Moreover, it has been recently shown that a polar filter, interacting with the 3′-OH and the triphosphate moiety of the incoming nucleotide, makes the 2′-OH of an rNTP clash with the surface of the fingers domain, limiting the possibility to bind rNTPs in a catalytically competent conformation [12]. The steric and the polar filters fall on nearly perpendicular planes, cooperating for elevated sugar selectivity [12].

However, sugar selectivity is not stringent enough, especially considering that DNA polymerases are constantly challenged by high rNTP concentrations. For example, even if in *Saccharomyces cerevisiae,* the dNTP pools increase of about three-fold upon entry into the S phase respect to G1 [13], and high ribonucleotide reductase (RNR) activity is maintained throughout the S phase [14], the physiological concentrations of the four rNTPs greatly exceed those of dNTPs [15,16]: rNTPs in yeast cells range from 500 to 3000 μM, while dNTPs are in between 12 and 30 μM, with rNTP:dNTP ratios varying from 36:1 for cytosine to 190:1 for adenine [16]. For this reason, pol ε has been estimated to introduce 1 rNMP every 1250 deoxyribonucleoside monophosphates (dNMPs) during leading strand synthesis, while pol δ and pol α, responsible for lagging strand synthesis [17], account for the incorporation of 1 rNMP every 5000 dNMPs and 625 dNMPs, respectively, resulting in more than 13,000 rNMPs inserted into the yeast genome for each replication cycle [16] (Table 1). Such high numbers also result from the reduced ability of pol ε and especially pol δ to proofread rNMPs inserted in DNA [18,19,20]. Ribonucleotides can thus be considered as the most common non-canonical nucleotides present in the eukaryotic genome [16,21]. The presence of ribonucleotides into genomic DNA has been confirmed in vivo by alkali-sensitivity assays [17], and subsequent studies revealed that the mean frequency of incorporation might be even higher, about 1 rNMP every 700 dNMPs [21]. Single or di-ribonucleotides have been detected in vivo also in mammalian genomic DNA and estimated to generate at least 1,000,000 alkali-sensitive sites per cell [22]. Additionally, different mutations in the active site of the three yeast replicative polymerases, which impact on their sugar selectivity, even induce higher frequencies of rNMPs incorporation. For example, for particular pol ε (*pol2-M644G*), pol δ (*pol3-L612M*), and pol α (*pol1-L868M*) variants, the rNMPs incorporation rate increases 10, 8, and 15 times, respectively [17,23,24].

### 2.2. Reparative DNA Synthesis

The activity of pol ε and pol δ is not only restricted to DNA replication. They are indeed involved in repair processes requiring DNA synthesis, in particular, nucleotide excision repair (NER) [41], so they may also introduce rNMPs in such circumstances. Reparative DNA synthesis steps are as well performed by many other specialized polymerases that can contribute to rNMPs incorporation (Table 1) [42].

The X-family polymerases pol β, pol λ, and pol μ are involved in base excision repair (BER), DSBs repair by nonhomologous end-joining (NHEJ), and specialized translesion synthesis (TLS) of oxidative lesions [43,44]. Pol β can place rNMPs opposite cyclobutane pyrimidine dimers (CPDs), and it is even able to synthesize stretches of up to eight rNMPs long in vitro [26]. Moreover, pol β (and, to a lesser extent, pol λ) can introduce ribonucleotides opposite 8-oxo-G lesions under physiological concentrations of metal activators and nucleotides [27]. Due to the lack of a steric gate residue, substituted by a single glycine residue [45], pol μ has a very low rNTPs/dNTPs discrimination rate [28], which allows it to insert rNMPs, promoting efficient DSBs repair by NHEJ [28,29,30]. The X-family Terminal deoxynucleotidyl transferase (TdT) has been long known to be important for the addition of template-independent nucleotides (N-regions) to gene segment junctions during V(D)J recombination [46,47], and it has as well only a minor preference for dNTPs over rNTPs in vitro, under conditions of in vivo rNTP/dNTP pool imbalance [31]. Y-family polymerases as pol η and pol ι are needed for TLS of many different types of DNA lesions [43,44]. The wild type *S. cerevisiae* pol η just shows a minimal rate of rNMP insertion on undamaged and damaged DNA; by contrast, the steric gate mutant *pol η-F35A* readily incorporates the correct rNMP opposite both templates, and in vivo experiments suggest that it may catalyze the incorporation of stretches of ribonucleotides in DNA [48,49]. Moreover, genetic evidence points towards the idea that under low dNTP conditions, either the wild type pol η or, even more, *pol η-F35A* inserts consecutive ribonucleotides, which become toxic in the absence of RNase H activity [34]. Differently from its yeast counterpart, the wild type human pol η inserts rNMPs opposite both undamaged and damaged DNA templates, even if maintaining base selectivity [32,33]. Human pol η can incorporate cytidine monophosphate (rCMP) opposite guanine, CPDs, 8-oxo-Gs, 8-methyl-2′-deoxyGs, and cisplatin intra-strand guanine crosslinks (cis-PtGG), and it is also capable of synthesizing polyribonucleotide chains [32,33]. The low sugar selectivity of human pol η may result not only by its extraordinarily spacious active site but also by the absence of the polar filter described above [12]. The human pol ι incorporates and extends ribonucleotides opposite damaged and undamaged bases depending on the sequence context [35]. Contrary to pol η, pol ι readily incorporates rNMPs also opposite abasic sites [35]. The A-family pol θ is a fundamental player in DSBs repair by alternative end-joining (alt-EJ) [50,51]. In vitro studies have demonstrated that, in the presence of Mn^2+^, pol θ has a robust template-independent terminal transferase activity and it is prone to incorporate rNMPs; this is intriguing, considering that Mn^2+^ is used by the MRX/MRN complex when generating 3′ ssDNA overhangs, which are the substrates of pol θ during alt-EJ [39]. Finally, the DNA-directed primase-polymerase PrimPol, belonging to the archaeo-eukaryotic primase superfamily, is able to use both rNTPs and dNTPs during replication initiation and chain elongation, when activated by Mn^2+^ (the preferred metal cofactor), as well as during the bypass of DNA lesions, even increasing the fidelity of synthesis opposite 8-oxo-G lesions [40]. Interestingly, rCMP paired opposite to damaged templates makes the RNase H2-dependent removal greatly inefficient. This may contribute to the accumulation of rCMP into genomic DNA [33], and it also seems to reduce the efficiency of the human OGG1 and MutYH base excision repair (BER) proteins [52], which may lead to a lack of 8-oxo-Gs removal, resulting in increased mutagenesis.

It should be emphasized that these polymerases are often active outside of the S phase [53,54,55,56] when the concentration of dNTPs is even lower than in the S phase [13], which may contribute to more significant incorporation of rNMPs into DNA. We can then speculate that “non-replicative” ribonucleotides may become particularly relevant in post-mitotic cells, such as neurons, where TLS has been recently found to take place [57].

## 3. Mechanisms of Single Ribonucleotides Removal

The high number of rNMPs incorporated into DNA, together with the observation that steric gate mutations, making replicative polymerases more stringent for sugar discrimination [17], have not been selected through the evolution, suggests that they must have some physiologic meaning. For example, two separate groups have demonstrated how rNMPs provide sites where the genomic DNA can be incised, allowing the mismatch repair machinery to be loaded onto the otherwise continuous leading strand in eukaryotic cells [23,58]. Single chromosome-embedded rNMPs must be anyway promptly removed, as their persistence has several negative consequences. Ribonucleotides left in DNA alter the shape and the conformation of DNA molecules [59,60,61,62], the assembly of nucleosomes [63,64], and they may hamper DNA replication since replicative DNA polymerases ε and δ are not efficient in bypassing them [16,18,19,65,66,67,68]. However, the most detrimental effects of single rNMPs seem to derive from their improper repair, as reviewed in [3].

To restore the correct DNA:DNA composition, cells have evolved ribonucleases H (RNases H), specialized in the removal of ribonucleotides from DNA. In eukaryotic cells, RNase H2 is composed of three subunits (Rnh201, Rnh202, Rnh203 in yeast; RNaseH2A, RNaseH2B, RNaseH2C in higher eukaryotes), all essential for the activity of the complex, and it cleaves both single and multiple rNMPs paired with DNA [69]. RNase H2 is the initiator of ribonucleotide excision repair (RER), the most common repair pathway for the removal of genomic embedded rNMPs [21]. RER ensures genome integrity and proper development of mouse embryos [22], keeping embedded rNMPs under a threshold of ribonucleotide tolerance [70]. In yeast, the main alternative strategy for processing ribonucleotides in DNA in the case of a faulty RER is based on Topoisomerase 1 (Top1) [71]. Top1-mediated mechanisms act mainly on the leading strand [72] and create unligatable 2′,3′-cyclic phosphate ends [73], which may have mutagenic effects [17,74,75], even resulting in DSBs [76]. Similarly, human Top1 can recognize and incise the DNA at the level of unrepaired rNMPs in RER-defective RNase H2-mutated human cell lines [77]. RNase H2 mutations are associated with a rare autoinflammatory disorder known as Aicardi–Goutières syndrome (AGS) [78], mainly characterized by early-age onset and chronic overproduction of type I interferon in the absence of infections [79]. Patient-derived cells accumulate rNMPs in their genome and exhibit constitutive post-replication repair (PRR) and DNA damage checkpoint activation [68,80]. The mechanism by which RNase H2 aberrations trigger the disease is still unclear, although over 50% of the studied AGS families are affected by mutations in one of the three RNase H2 genes [81,82]. Moreover, RNase H2 dysfunctions have also been associated with some types of cancer [83,84,85,86,87] and with systemic lupus erythematosus (SLE) [80].

Eukaryotic cells also possess another specialized ribonuclease H, RNase H1, which is a single subunit protein that cleaves stretches of at least four consecutive rNMPs. Its enzymatic activity is essential for mitochondrial DNA replication in mammals [88], while it does not seem to be required during RER [21].

## 4. Multiple rNMPs Embedded into DNA: One Possible Cause of Genome Instability and Cell Lethality

Although the presence of single ribonucleotides into the chromosomal DNA has been extensively investigated in many organisms, whether the incorporation of consecutive rNMPs is also possible is still unclear. Unlike single rNMPs, which are moderately tolerated up to a certain threshold, multiple rNMPs might be even more detrimental for cellular viability. Indeed, even a few consecutive rNMPs can represent an insuperable obstacle during DNA replication because they cannot be correctly copied by the replicative DNA polymerases δ and ε that progressively stall when encountering four or more rNMPs [66,67]. A similar effect has been observed in mammalian mitochondria, where only RNase H1 activity is present: if multiple rNMPs embedded in mitochondrial DNA (mtDNA) are not properly removed, they cause a block of the replication fork, resulting in breakdown and loss of mtDNA [89]. Additionally, multiple rNMPs in the nucleus of *S. cerevisiae* cells are only tolerated, thanks to the action of the two main pathways of PRR: template-switch and TLS pol ζ [66]. Finally, similarly to single rNMPs, but even more significantly, polyribonucleotide chains may alter the proper conformation of DNA [59,60,62] and interfere with protein binding [63,64], possibly causing catastrophic defects in chromosome segregation and a global alteration of gene expression profiles. For all these reasons, further investigation of multiple rNMPs’ metabolism appears very important. 

**Figure 1:**
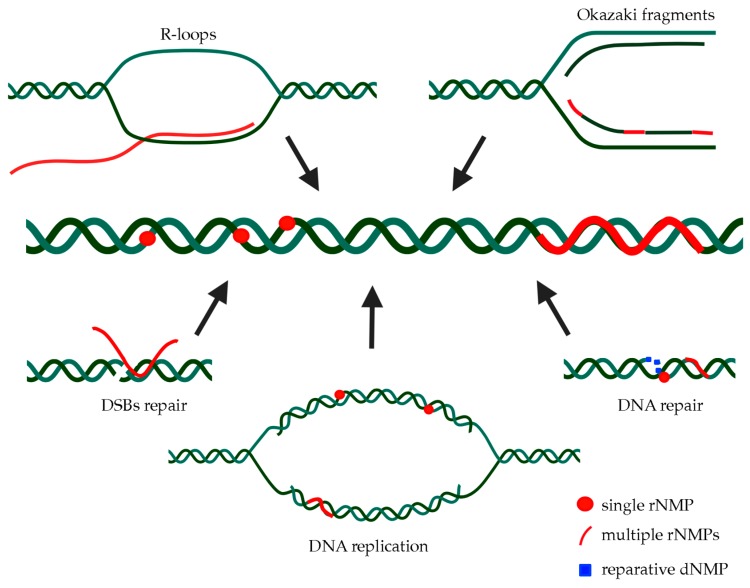
Sources and forms of rNMPs embedded in genomic DNA. Single ribonucleotides are primarily introduced in DNA by several DNA polymerases carrying out genome duplication and/or reparative DNA synthesis; their activity may also result in the direct incorporation of polyribonucleotide chains. Stretches of consecutive ribonucleotides embedded in chromosomal DNA may also derive from the aberrant processing of RNA:DNA hybrid structures, like RNA primers required for Okazaki fragments’ synthesis, R-loops, and hybrids at double-strand breaks (DSBs) sites.

Unfortunately, the study of multiple embedded rNMPs is complicated by the fact that it requires the simultaneous removal of RNase H1 and RNase H2, which can both recognize stretches of more than four consecutive rNMPs. *S. cerevisiae* represents an excellent model organism to this purpose because mutants lacking all RNase H activities are still viable [66]. Nevertheless, RNases H can potentially process any polyribonucleotide tract in DNA (stretches of rNMPs, R-loops, RNA primers found at Okazaki fragments, etc.), so it remains difficult to establish which one of these unprocessed substrates causes the observed effects. Anyway, if stretches of consecutive rNMPs do exist, how they are incorporated (Figure 1) and subsequently removed needs to be clarified. We have discussed below the different possible sources of multiple embedded rNMPs.

### 4.1. DNA Polymerases

Despite DNA polymerases being primarily responsible for the incorporation of single rNMPs, only mutant variants seem capable of introducing consecutive rNMPs. The pol ε variant *pol2-M644G* mentioned above incorporates rNMPs in DNA at higher frequencies than the wild type pol ε [17]. The fact that this mutant becomes synthetic lethal with the simultaneous absence of RNase H1 and H2 suggests that it incorporates stretches of rNMPs, requiring the activity of both RNases H to be removed [24,66]. On the contrary, pol α and δ variants that incorporate more rNMPs are still viable when combined with RNase H1 and H2 mutants [24]. This could be explained by a low rNMPs density in the lagging strand, possibly correlating with a low probability of introducing consecutive rNMPs [24]. Alternatively, RNase H independent mechanisms may remove single and multiple rNMPs, when incorporated in the discontinuous lagging strand [24].

As already discussed, also the *S. cerevisiae polη-F35A* steric-gate mutant seems to incorporate polyribonucleotide tracts in DNA at a high rate, leaving a specific 1 bp deletion signature, when not removed by RNase H2 [48,49]. Moreover, under particular stress conditions, also wild type replicative and/or reparative DNA polymerases may incorporate consecutive rNMPs. This is what has been suggested for the *S. cerevisiae* pol η. Meroni et al. found that, upon replication stress induced by hydroxyurea, pol η was recruited at stalled replication forks, where it facilitated the formation of stretches of rNMPs that became highly toxic for cells, when not properly replaced with DNA [34].

### 4.2. Okazaki Fragments 

Although the number of rNMPs incorporated during DNA replication is surprisingly large, the main source of genomic ribonucleotides remains by far the replication priming. Replicative DNA polymerases require a piece of RNA initiator (RNAi) of ~8–10 nt in length to properly work and replicate DNA. Considering the discontinuous nature of the lagging strand, this is translated in an average of ~100,000 RNA:DNA hybrids formed at each round of DNA replication in *S. cerevisiae* and in more than 10 millions of hybrids found in human cells [90,91]. RNA:DNA primers must then be removed, and Okazaki fragments (OKFs) joined together, forming a continuous lagging strand. Because of their abundance, it is easy to imagine how just a few defects in their processing may have deleterious consequences in cells. Different pathways cooperate in Okazaki fragments maturation (reviewed in [92]). The dominant pathway seems to be dependent on FEN1 (Rad27 in *S. cerevisiae*), with the additional contribution of Exo1 cleaving the short flaps (2–10 nt in length), generated when the RNAi is displaced through pol δ-mediated DNA synthesis [93,94,95]. When flaps become longer (>30 nt), the ssDNA generated is coated by RPA, which inhibits the activity of Fen1; the processing of such intermediates requires Dna2 activity [96]. When strand displacement does not occur, also RNase H2 seems to have a role in the direct hydrolysis of RNA:DNA primers [97]. *S. cerevisiae* strains, lacking Rad27 and RNase H2, are sick but become lethal when combined with RNase H1 deletion. This seems to suggest that, besides RNase H2, also RNase H1 has a role in Okazaki Fragments maturation [98]. Finally, the generated nicks are sealed by DNA Ligase I (Cdc9 in *S. cerevisiae*) [99]. The exact composition, crosstalk, and regulation of all these pathways are still largely unknown, but dysfunctions in any of these mechanisms could leave flaps or nicks into the genome, causing deletions, amplification of DNA sequences, and DSBs [100]. Moreover, even if never visualized, dysfunctions could also result in the stable inclusion of RNA stretches into DNA, as suggested by different groups [34,101]. Intriguingly, Holmes et al. [89] found that this also happened in the mouse mitochondrial genome, where, in the absence of RNase H1, the RNA primers were fixed in both template strands of mtDNA, causing dramatic effects on mtDNA replication. The incorporation of an RNA primer into the DNA is also the proposed mechanism for mating-type switching in *Schizosaccharomyces pombe.* During the S phase, two consecutive rNMPs are left by incomplete processing of RNA primer into the lagging strand at the *MAT1* locus; these rNMPs are maintained until the following replication cycle, inducing polymerase stalling, and recombination events, which lead to mating-type switching [102,103].

### 4.3. R-Loops

Another important source of ribonucleotides in DNA is represented by R-loops, peculiar three-stranded structures formed when a transcribed RNA hybridizes back to the template, leaving the non-template DNA single-stranded [104]. These hybrid regions are longer than the canonical 8 bp hybrids formed by active RNA polymerases (RNAPs) [105], and R-loop-prone regions cover about 8% of the yeast genome [106]. Growing evidence suggests that these structures play important roles in regulating gene expression [107] and chromatin structures [108]. On the other hand, they can compromise genome integrity since R-loops expose patches of ssDNA, which are more susceptible to mutagenesis, recombination, and DNA damage, compared to dsDNA (reviewed in [109]). Moreover, conflicts between the DNA replication machinery and R-loops trigger fork collapse and DSBs [110,111]. Tight R-loop homeostasis must thus be maintained in cells, to prevent their negative outcomes while maintaining positive functions. Understanding how this regulation occurs is a big challenge, and, to date, many factors have been identified as important ones for preventing, resolving, but also promoting R-loops formation (reviewed in [112,113]). 

The formation of R-loops is prevented by mRNA biogenesis and processing proteins that reduce the ability of RNA transcripts to re-hybridize with the DNA behind RNAPs [114,115] and by DNA topoisomerases that relax negative supercoils formed behind the transcriptional bubble [116,117]. Once formed, different factors can act to remove R-loops, like RNase H enzymes (H1 and H2), which cleave the RNA moiety of RNA:DNA hybrids [69] and numerous helicases that unwind the hybrids, as Senataxin (Sen1 in *S. cerevisiae*) [118], the human DHX9 [119], and Pif1-family helicases [120]. Rad51, instead, seems to actively promote R-loops formation [121].

Different situations have been described where the RNA stretch present into R-loops becomes embedded into DNA. In prokaryotic cells, R-loops are frequently associated with origin-independent replication [122,123]. In vitro studies have shown that prokaryotic DNA polymerases can use mRNA as a primer when the replication fork collides with the RNA polymerase [124], and this is also the case for eukaryotic cells. Stuckey et al. [125] found that in *S. cerevisiae,* RNA polymerase I transcription constraints led to persistent R-loops in the ribosomal DNA locus. Here, the RNA present in the R-loop can be used as a primer by DNA polymerases, triggering an origin-independent replication process. Being highly inaccurate, this unscheduled replication can cause genome instability.

### 4.4. Hybrids at DSBs

The local incorporation of ribonucleotides and the presence of different types of RNA molecules have been shown to have important effects even on DNA DSBs, influencing their repair by nonhomologous end-joining or homologous recombination pathways (reviewed in [126,127,128]). For example, Pryor et al. recently reported that one or more rNMPs were transiently incorporated at broken DNA ends by pol μ or TdT, enhancing DSB repair by NHEJ mechanisms [30]. Growing evidence shows that also the hybridization of complementary RNA molecules at DSB ends regulates their repair (reviewed in [126,127,128,129]); different groups have indeed observed an accumulation of RNA:DNA hybrids at DSB sites [130,131,132,133,134,135,136,137,138,139,140]. The origin of such RNA species is still under investigation. One possibility is that, after DNA damage, RNA polymerase II is recruited at the broken ends, generating newly transcribed RNA, as suggested in [130,132,139,141]. An alternative, which can coexist with the former mechanism, is that the RNA molecules may result from transcripts produced before the formation of the break in active genes [133,134,137,138]. Regardless of the source of RNA:DNA hybrids, the most discussed point is the understanding of their significance when repairing DSBs. Notably, RNA:DNA hybrids seem to contribute to the recruitment of repair factors [131,132,133,134,135,136,137,138,139,140] and to the control of DNA end resection [132,137,138,139], the fundamental process creating 3′ end ssDNA filaments needed for recombination [142]. However, how RNA:DNA hybrids impact on DSB processing and repair is still an open debate [143]. Indeed, while some data indicate that they promote resection [136,137,139], others suggest an anti-resection role [132,138] or no effect at all [134]. More work is thus required to clarify the regulation of this dynamic phenomenon. Furthermore, it has long been known that DSBs repair can proceed through the formation of a cDNA intermediate [144,145]. Perhaps related to those early observations, it has also been discovered that, when RNase H enzymes are not functional, endogenous RNA itself can directly be used as a template for DSBs repair [129].

In conclusion, even if there is now a large body of evidence showing that RNA:DNA hybrids participate in DSBs repair, many aspects should be investigated and defined. Moreover, as mentioned for R-loops, and RNA primers at Okazaki fragments (OFs), it is tempting to speculate that, also in the context of DSBs repair, improperly removed RNA tracts might remain embedded at DSB ends, posing a threat to genome stability.

## 5. Mechanisms of Multiple Embedded Ribonucleotides Removal

Once defined the different processes that could generate tracts of rNMPs embedded into DNA, the question that arises is: how are these substrates processed in cells? As previously mentioned, single rNMPs are the substrate of RER [21], but whether this pathway also works on multiple rNMPs has never been proved. It is unlikely that the pathways acting on R-loops and OFs could process multiple rNMPs, once embedded into DNA, and thus inaccessible to players like helicases. Since RNase H1 and H2 both process consecutive embedded rNMPs, they represent the main candidates for their removal. Anyway, how the two enzymes work in vivo on these structures needs further clarification. Some progress has been made, thanks to the development of a separation-of-function mutant of the RNase H2 enzyme, called *rnh201-RED* (ribonucleotide excision defective), which loses the ability to remove single rNMPs, but retains a discrete activity on consecutive rNMPs [146]. This mutant has been extremely useful to enlighten the role of the two functions of RNase H2 (reviewed in [147]). Being still able to remove multiple rNMPs, the *rnh201-RED* mutant alone cannot prove their existence; the development of additional separation-of-function mutants may thus be useful.

## 6. Ribonucleotides into the DNA of Organelles

Besides being present into the nuclear DNA, ribonucleotides are also found in the DNA contained in two types of eukaryotic organelles: mitochondria [148,149,150] and chloroplasts [151].

The human mitochondrial DNA (mtDNA) is a circular multicopy molecule of 16.5 kb, composed of two filaments, named heavy (H) strand and light (L) strand, and whose replication mechanism is not completely resolved. Different models for mitochondrial DNA duplication have been proposed, which are well described in recent reviews [152,153]; here, we only summarized the types and the sources of rNMPs that could be found into mtDNA (reviewed in [154]).

Replication primers represent the first source of consecutive rNMPs also in mtDNA. However, they seem to be synthesized by the mitochondrial RNA polymerase POLRMT and not by a replicative primase, as it happens for the nuclear DNA [155]. Such transcripts are stabilized by G-quadruplex structures formed in the non-template DNA strand, resulting in mitochondrial R-loops that act as replication primers [156]. Polyribonucleotide chains could also result from long RNA transcripts, which temporally coat the displaced H-strand, generating RNA:DNA hybrids that function as lagging strands during mtDNA replication, as proposed by one of the models used to explain mtDNA replication called RITOLS (ribonucleotide incorporation throughout the lagging strand). These long RNAs may result from a primase activity or by the hybridization of the displaced DNA with preformed RNA transcripts [157]. RNase H1 is the factor responsible for the removal of multiple rNMPs from mtDNA. The mammalian RNase H1 is recruited into the organelles, thanks to an essential mitochondrial localization domain, and failures in its activity cause mitochondrial dysfunctions. In mouse, when RNase H1 is absent, replication primers are not properly removed, and stretches of RNA remain fixed in both template strands of mtDNA [89]. This is a perfect example of how tracts of embedded rNMPs can compromise genome integrity. Since they cannot be bypassed by the mtDNA polymerase γ, they lead to persistent DNA gaps that are catastrophic for the subsequent round of replication [89]. As a consequence, mice lacking RNase H1 die during embryogenesis [88]. In humans, mutations in RNase H1 have been associated with mitochondrial encephalomyopathy with adult-onset [158]. These examples highlight the importance of removing multiple rNMPs from mtDNA.

**Figure 2:**
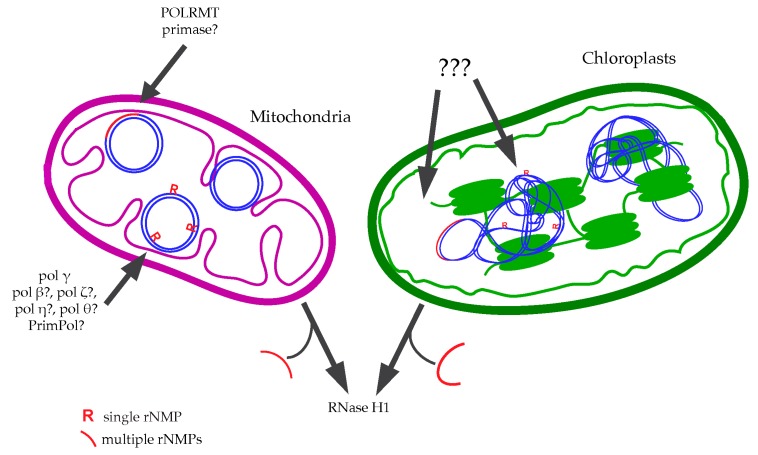
rNMPs incorporation and removal from mitochondrial and chloroplast DNA. Single and multiple ribonucleotides found into the DNA of mitochondria and chloroplasts may result from different sources. Several DNA polymerases might contribute to the incorporation of rNMPs, as demonstrated for pol γ acting on mtDNA. The activity of RNase H1 is essential for processing polyribonucleotide chains synthesized for replication priming, while single rNMPs remain unprocessed due to the absence of RNase H2.

Besides stretches of rNMPs, single ribonucleotides are as well incorporated during mtDNA replication. Intriguingly, unlike the nucleus, mitochondria completely lack RNase H2 or other mechanisms for the removal of single rNMPs [159]. As a result, it has been estimated that 30–60 rNMPs persist in each mtDNA molecule of different human and mouse cell lines [38,160]. rNMPs have been mapped in these cells, revealing that they have a random distribution, no strand specificity, and that rAMP is the most frequently found [160,161]. These few single rNMPs may result by the action of the replicative DNA polymerase γ responsible for mtDNA duplication, despite its efficiency in discriminating against rNTPs and in the bypass of previously incorporated rNMPs [37,38]. Anyway, other DNA polymerases seem to contribute to mtDNA replication after DNA damage, like PrimPol [162] pol β, pol ζ, pol η, and pol θ (reviewed in [163]); thus, we cannot exclude a minor contribution of these latter ones in rNMPs incorporation (Figure 2).

Ribonucleotides have also been observed into the DNA of chloroplasts, the other organelles capable of autonomous replication in plant cells. The chloroplast DNA (cpDNA) consists of linear or circular multicopy molecules of 120–170 kb, which can replicate in different manners (reviewed in [164]). Even if there is still much to learn about rNMPs in the DNA of chloroplasts, it is evident that stretches of multiple rNMPs can compromise cpDNA stability. Apart from RNA tracts used for DNA replication priming, R-loops can be frequently found in these organelles. It has been found that the AtRNaseH1-like protein (RNH1C), together with DNA gyrases, plays a key role in the processing of these hybrids, maintaining chloroplast DNA integrity [165,166]. In addition, also single rNMPs have been observed into the cpDNA of some species of plants, with an estimation of 12–18 rNMPs per molecule [151] (Figure 2). However, the origin, location, and significance of their presence are still unknown, as well as the existence of RNase H2-like enzymes able to remove these structures.

Although rNMPs in mtDNA and cpDNA need to be further explored, their existence in these endosymbiotic organelles is extremely intriguing. This “incorrect” sugar selection comes from ancestral forms of life and is conserved in evolved organisms, suggesting that they have been maintained throughout the evolution to perform physiological functions.

## 7. Methods to Map and Quantify Ribonucleotides in DNA

**Figure 3:**
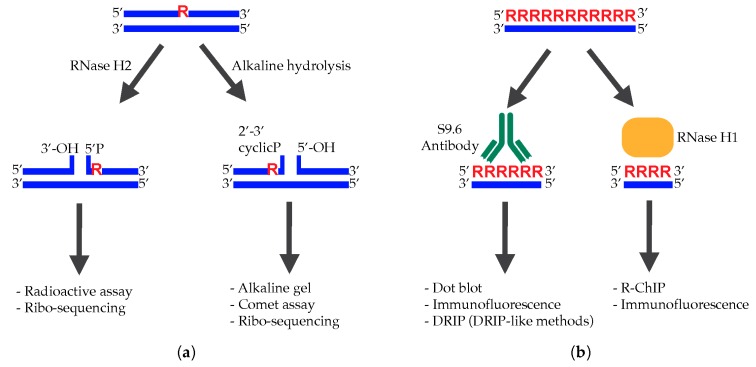
Currently available techniques for studying RNA in DNA. (**a**) The investigation of single ribonucleotides paired with DNA is based on enzymatic or chemical digestion in correspondence of rNMPs to generate single breaks in DNA. Different approaches can then be used to visualize and map the sites of rNMP insertion. (**b**) The main strategies developed to examine stretches of ribonucleotides hybridized with DNA rely on the S9.6 monoclonal antibody or on RNase H1, which allows recognizing RNA:DNA hybrids independently of their sequence. S9.6 binds DNA hybrids of at least 6 bp; RNase H1 detects hybrids of at least 4 bp.

At this point, it is clear that RNA can hybridize to DNA in different ways and under different forms, having beneficial but also detrimental effects in cells. It is, therefore, crucial to study and map these structures with precise, quantitative, and reproducible techniques. We have concluded our review with an overview of the most common strategies available for studying in vivo, either single or stretches of rNMPs hybridized with DNA; strong and weak aspects of each method are indicated (Figure 3).

### 7.1. Single rNMPs Paired with DNA

As mentioned above, the highly reactive 2′-OH group present on the ribose ring of ribonucleotides can attack the adjacent phosphodiester bonds, generating breaks by alkaline hydrolysis [5]. In the presence of a basic solution, the genomic DNA is, therefore, nicked in correspondence of embedded rNMPs, originating fragments that can then be visualized by staining with SYBR Gold or other DNA intercalating dyes, after electrophoresis in alkaline conditions [16,22]. The average size of the fragments correlates with the frequency of rNMPs introduction. Besides this global indication, it is also possible to selectively probe ribonucleotides incorporated into specific regions by Southern blot analysis after digestion with appropriate enzymes. Furthermore, using a strand-specific probe, it is possible to discriminate ribonucleotides incorporated into the leading or lagging replicated-strand [167,168,169]. Although the alkaline electrophoresis-based approach is widely used, it is very hard to understand whether fragments are exclusively due to embedded rNMPs. Nicks/gaps caused by incomplete replication or nicks generated during DNA manipulation cause the same fragmentation in denaturing conditions. These experiments should, indeed, always be compared with a denaturing condition that does not affect the hydrolysis of ribonucleotides [22].

Similarly, comet assay has also been adapted to measure ribonucleotides embedded into the DNA of human and mouse fibroblasts, as well as in cells collected from patients with SLE and AGS [80]. After nicking the genomic DNA with the bacterial RNase HII, electrophoresis is performed in an alkaline buffer. The migration of the fragmented DNA leads to the formation of comets visualized by fluorescent microscopy after SYBR Gold staining. The length and intensity of the comet tail are proportional to the level of ribonucleotides [170]. Compared to alkaline electrophoresis, the manipulation of the sample is minimal, making the result more reproducible. However, even this technique does not allow distinguishing nicks/gaps from rNMPs.

Hiller et al. [171] were the first to describe another extensively used approach subsequently applied by other groups [68,172]. After extraction, the genomic DNA is treated in vitro with the bacterial RNase HII, which introduces nicks at every site of ribonucleotide incorporation. These nicks are then radioactively labeled, taking advantage of the DNA polymerase I nick translation capability. The radioactive signal reflects the level of genomic ribonucleotides. With this approach, the advantage is that a comparison of the signals-obtained +/− RNase HII digestion allows discriminating between ribonucleotide-dependent nicks and nicks generated during DNA preparation.

The main limitation of all these approaches, however, is that they are only semi-quantitative and probably only sensitive enough to detect big changes in the ribonucleotide content. Moreover, they sometimes give inconsistent results.

High-throughput sequencing techniques bypassed these limitations, allowing the study of embedded rNMPs with single-nucleotide resolution. This was made possible, thanks to the development of four different strategies: embedded ribonucleotide sequencing (emRiboSeq) [173], hydrolytic end-sequencing (HydEn-seq) [174], ribose-seq [20], and polymerase usage sequencing (Pu-seq) [175]. The genomic DNA is extracted from RNaseH2-defective strains, and it is nicked in the correspondence of the embedded rNMPs. This can be done either enzymatically with RNase H2 [173], or chemically by exploiting alkaline hydrolysis [20,174,175]. Fragments are then ligated to adaptors and sequenced by next generation sequencing (NGS) approaches. Independently of the technique used, raw sequencing data can be analyzed using a novel open-sources software (http://github.com/agombolay/ribose-map) [176]. A similar approach is RADAR-seq (rare damage and repair sequencing) [177]. Here, nicks generated by RNaseH2 are replaced with a patch of modified bases, thanks to a nick translation reaction. The detection of such modified bases by PacBio single molecule, real-time (SMRT) sequencing reveals the location of ribonucleotides [177]. Moreover, by using steric-gate mutants, which incorporate more rNMPs, it has been even possible to assess the precise contribution of replicative and TLS polymerases to DNA replication [20,49,173,174,177]. To date, these approaches have been used in bacteria, archaea, and yeast cells, but they could be adapted to any organism in which RNase H activity can be modulated. They have allowed demonstrating that the rNMPs distribution is non-random and that mitochondrial DNA, Ty regions, and rDNA locus are preferential hotspots [20]. However, all the experiments have been performed in RNase H-deficient strains, where every replication round occurs in the presence of thousands of rNMPs accumulated in the DNA template, which compromises the progression and fidelity of DNA polymerases [19,66]. This could have an influence on the incorporation of rNMPs, masking the real hotspots introduced in a single round of DNA replication. The use of an RNase H conditional mutant [34], which can be switched off just prior to entering the S phase, could be a useful strategy to map the unaffected positions of rNMPs.

Overall, all the strategies described until now exploit the same principle: enzymatic or chemical digestion in correspondence of rNMPs, to generate a single break. This makes it impossible to discriminate between one or several consecutive rNMPs. Indeed, the presence of stretches of embedded ribonucleotides has never been observed. One possibility could be to extract the genomic DNA of RNase H-defective cells and incise only multiple rNMPs with RNase H1 or *RNase H2-RED* [146]. Ribose-sequencing approaches can then be applied. This should avoid the high signal generated by single rNMPs that might mask the signal due to just a few stretches of embedded ribonucleotides.

### 7.2. Stretches of rNMPs Hybridized with DNA

The main strategies available at the moment to detect multiple ribonucleotides hybridized to DNA rely on the use of the S9.6 monoclonal antibody or on a catalytically inactive version of RNase H1 (reviewed in [178]). Although these tools are massively used to study R-loops, we have to keep in mind that they can recognize any hybrid present in the genome: e.g., R-loops, DNA replication primers, stretches of embedded ribonucleotides, hybrids at DSBs. Moreover, even if with lower affinity, both S9.6 and RNase H1 can also recognize RNA:RNA hybrids [179,180]. In particular, S9.6 binds RNA:DNA hybrids with at least six consecutive ribonucleotides [179], even if the binding affinity seems to be influenced by the sequence context [181]. In addition to S9.6 antibodies, RNA:DNA hybrids can be detected by using the RNase H1 N-terminal hybrid-binding domain (HDB), which can even recognize stretches made up by just four ribonucleotides [182]. Finally, D5H6 is another antibody able to react with RNA:DNA hybrids [183,184], even if less efficiently, compared to the other systems. Independently of the used tools, treatment with RNase H1 is then essential to prove that the signal obtained is specific for RNA:DNA hybrids.

A first indication about the global level of hybrids present in the genome can be obtained by a dot blot assay [165,185,186,187], where serial dilutions of genomic DNA are spotted on a membrane and subsequently hybridized with S9.6. Indications about the abundance and localization of RNA:DNA hybrids can also be obtained by immunofluorescence studies. The S9.6 antibody has been extensively used for this purpose [183,188], while Aguilera and colleagues used the HBD of RNase H1 fused with the green fluorescent protein (GFP), forming the so-called HB-GFP [188]. Both these strategies led to the identification of RNA:DNA hybrids in the nucleus of cells, with high intensities detected in the nucleolar region (where the majority of R-loops are formed [117]), as well as in the cytoplasm, possibly because of the abundant RNA:DNA hybrids present in mitochondria.

DNA-RNA immunoprecipitation (DRIP) is currently the most used and accurate technique for mapping genomic RNA:DNA hybrids. It was initially described by the Tollervey’s lab [117], and, since then, many variations have been developed (S1-DRIP, bisDRIP, DRIPc, ssDRIP, etc.) [106,189,190,191]. After chromatin extraction and fragmentation, RNA:DNA hybrids are immunoprecipitated with the S9.6 antibody. The precipitated material is then purified and used for rtPCR reactions, or sequenced, to study the genome-wide distribution of hybrids (DRIP-seq). R-ChIP is a similar approach that uses a catalytically inactive RNase H1, which can still bind hybrids [192,193]. However, the resolution of these techniques depends on the dimension of the immunoprecipitated DNA fragments, and the results obtained are not always reliable. Moreover, probably due to the big number of different protocols available, the results obtained by different groups are sometimes contrasting [194]. Nevertheless, to date, DRIP is considered as the most accurate system to detect and map RNA:DNA hybrids. We have to remember, however, that the latter does not include only R-loops, but any structure in which stretches of RNA anneal to DNA.

## 8. Concluding Remarks

Although stretches of multiple embedded rNMPs have only been observed in mtDNA, their presence in the nuclear DNA has also been genetically predicted. The persistence of multiple rNMPs in the mitochondrial DNA has been shown to have detrimental effects, and so is suspected for genome-embedded polyribonucleotide chains, with consequences even more severe than those deriving from unprocessed single rNMPs. Different techniques are currently available to study single rNMPs and RNA:DNA hybrids, but further efforts should be made for the development of groundbreaking methods, allowing to isolate only the desired category of RNA:DNA hybrids, and to distinguish sites of single rNMPs insertion from sites with multiple rNMPs. Demonstrating the existence of consecutive embedded rNMPs, and discovering details about their sources and removal, might help to clarify the contribution of the two RNases H in the recognition and processing of all hybrid structures and, importantly, to shed light on the mechanisms linking RNA:DNA hybrid structures, replication stress, genome instability, and severe human pathologies.

## Figures and Tables

**Table 1:** Ribonucleoside monophosphates (rNMPs) insertion by eukaryotic DNA polymerases opposite different DNA templates. Eukaryotic DNA polymerases are classified according to family type and roles in DNA transactions; their ability to synthesize ribonucleotides opposite different types of DNA templates is then reported.

Who	Family	Role In	rNMPs Insertion
pol ε	B	replication/repair	undamaged leading strand [16]
pol δ	B	replication/repair	undamaged lagging strand [16]
pol α	B	replication/repair	undamaged lagging strand [16]
pol ζ	B	translesion synthesis (TLS); mitochondrial replication	rare [25]
pol β	X	repair/TLS	undamaged template, CPDs [26] 8-oxo-Gs [27]
pol λ	X	repair/TLS	8-oxo-Gs [27]
pol μ	X	repair	NHEJ ends [28,29,30]
Tdt	X	repair	N-regions of V(D)J ends [31]
pol η	Y	TLS; lesion-independent replication stress	undamaged template [32,33,34]; 8-oxo-Gs, CPDs, cis-PtGG, 8-methyl-2′-deoxyGs [32,33]
pol ι	Y	TLS	undamaged template, 8-oxo-Gs, abasic sites [35]
pol κ	Y	TLS	unknown
Rev1	Y	TLS	rare [36]
pol γ	A	mitochondrial replication	rare [37,38]
pol θ	A	TLS/repair	alt-EJ ends [39]
pol ν	A	TLS/repair	unknown
PrimPol	Archaeo- eukaryotic primase superfamily	priming/TLS	undamaged template, 8-oxo-Gs [40]

## Abbreviations

AGS	Aicardi–Goutières syndrome
BER	base excision repair
Bis-DRIP	bisulfite DNA:RNA immunoprecipitation
cpDNA	chloroplast DNA
CPDs	cyclobutane pyrimidine dimers
dNMP	deoxyribonucleoside monophosphate
dNTP	deoxyribonucleoside triphosphate
DRIP	DNA:RNA immunoprecipitation sequencing
DRIPc	DNA:RNA Immunoprecipitation followed by cDNA conversion
DSB	double-strand break
emRiboSeq	embedded ribonucleotide sequencing
GFP	green fluorescent protein
HBD	hybrid binding domain
HydEn-seq	hydrolytic end sequencing
mtDNA	mitochondrial DNA
NER	nucleotide excision repair
NHEJ	nonhomologous end-joining
OKFs	Okazaki fragments
PRR	post-replication repair
Pu-seq	polymerase usage sequencing
R-ChIP	R-loop chromatin immunoprecipitation
RADAR-seq	rare damage and repair sequencing
rDNA	ribosomal DNA
RED	ribonucleotide excision defective
RER	ribonucleotide excision repair
RITOLS	ribonucleotide incorporation throughout the lagging strand
RNAi	RNA initiator
RNAPs	RNA polymerases
RNase H	ribonuclease H
rNMP	ribonucleoside monophosphate
RNR	ribonucleotide reductase
rNTP	ribonucleoside triphosphate
S1-DRIP	S1 nuclease DNA:RNA immunoprecipitation
SLE	systemic lupus erythematosus
ssDNA	single-strand DNA
ssDRIP	ssDNA ligation-based library construction from DRIP
TdT	terminal deoxynucleotidyl transferase
TLS	translesion DNA synthesis
